# What outcomes should be evaluated in critically ill patients?

**DOI:** 10.5935/0103-507X.20210040

**Published:** 2021

**Authors:** Cassiano Teixeira, Marcelo Kern, Regis Goulart Rosa

**Affiliations:** 1 Departament of Internal Medicine, Postgraduate Program in Rehabilitation Sciences, Universidade Federal de Ciências da Saúde de Porto Alegre - Porto Alegre (RS), Brazil.; 2 Departament of Internal Medicine, Hospital Moinhos de Vento - Porto Alegre (RS), Brazil.; 3 Project Office, Hospital Moinhos de Vento - Porto Alegre (RS), Brazil.

**Keywords:** Patient outcome assessment, Critical illness, Critical care, Prognosis, Intensive care units, Avaliação de resultados da assistência ao paciente, Estado terminal, Cuidados críticos, Prognóstico, Unidades de terapia

## Abstract

Randomized clinical trials in intensive care prioritize disease-focused outcomes rather than patient-centered outcomes. A paradigm shift considering the evaluation of measures after hospital discharge and measures focused on quality of life and common symptoms, such as pain and dyspnea, could better reflect the wishes of patients and their families. However, barriers related to the systematization of the interpretation of these outcomes, the heterogeneity of measurement instruments and the greater difficulty in performing the studies, to date, seem to hinder this change. In addition, the joint participation of patients, families, researchers, and clinicians in the definition of study outcomes is not yet a reality.

## Abstract (pt)

Estudos clínicos randomizados em terapia intensiva priorizam desfechos focados em doença e não desfechos centrados no paciente. Uma mudança de paradigma considerando a avaliação de medidas após a alta hospitalar e medidas focadas na qualidade de vida e em sintomas comuns, como dor e dispneia, poderiam refletir melhor os desejos de pacientes e de seus familiares. No entanto, barreiras relacionadas à sistematização da interpretação desses desfechos, a heterogeneidade de instrumentos de medida e a maior dificuldade na execução dos estudos, até o momento, parecem dificultar essa mudança. Além disso, a participação conjunta de pacientes, familiares, pesquisadores e clínicos na definição dos desfechos dos estudos ainda não é uma realidade.

## INTRODUCTION

Conceptually, the emergence of intensive care units (ICUs) was based on the premise of saving the lives of critically ill patients, i.e., reducing mortality. This goal was achieved in many clinical situations, such as sepsis^(1)^ and acute respiratory failure.^(2)^ This was due to progress in vital organ monitoring techniques,^(3)^ to the organization and specialization of teams^(4-6)^ and to improvements in symptomatic treatments of multiple organ dysfunction syndrome (MODS).^(7)^

Although the goal - mortality - remains valuable for intensivists, patient-centered outcomes have gained importance over the years. Among these outcomes are more effective pain control, evaluation of medium- and long-term results in those who survive a critical illness and greater attention to caregivers and family members (new class of patients).^(8-10)^

Thus, the objective of this study is, through a narrative review, to describe the most important outcomes for critically ill patients, compare those with the outcomes most frequently studied in randomized clinical trials (RCTs) and describe possible barriers that prevent the evaluation of patient-centered outcomes in the intensive care setting.

## METHODS

This is a nonsystematic review in which the bibliographic references of the retrieved studies were also searched to identify other relevant studies.

The MEDLINE^®^ literature search was conducted in PubMed^®^ on August 25, 2020, using search terms and synonyms for “patient-centered outcome” and “critical illness”. Only articles found in Portuguese, English and Spanish were reviewed, totaling 40,629 abstracts published in the last 10 years. Based on these abstracts, articles were selected for the development of this study. The articles were reviewed for their contribution to the current understanding of the outcomes evaluated in ICU patients, with priority for reviews, meta-analyses, systematic reviews and RCTs.

### CURRENT FOCUS OF THE STUDY OF INTENSIVE CARE MEDICINE

The outcomes described in RCTs are distributed hierarchically.^(11)^ The researchers characterize mortality (category 1) and morbidity (category 2; for example: need for hospitalization, recurrence of disease, and need for dialysis therapy, among others) as the most important outcomes. The presence of symptoms (e.g., pain, dyspnea, and fatigue, among others), quality of life and functional status are classified in category 3, surpassing in importance only the substitute outcomes of category 4 (e.g., blood pressure, oxygenation level, and levels of interleukins, among others). These authors believe that the study of mortality is a consensual opinion between physicians and patients and therefore indisputable.^(9,12)^ In addition, other very important outcomes for physicians, such as the use of health resources or the duration of mechanical ventilation, seem to be much less important for patients when compared to the presence of pain or dyspnea.^(9)^ Thus, the answers that physicians seek when performing a clinical study are probably not the same as those that patients would like to obtain.^(9,12-14)^

**Figure 1:**
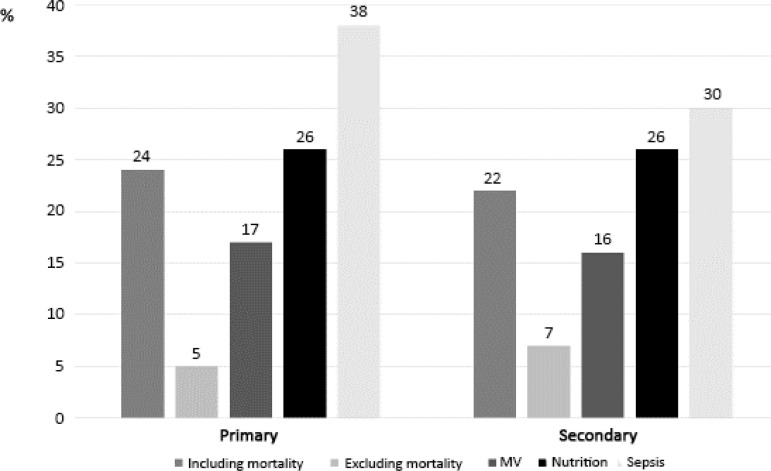
Prevalence of randomized clinical trials evaluating critically ill patients with primary or secondary patient-centered outcomes, as well as their distribution based on the focus of the study. The definitions “including mortality” and “excluding mortality” refer to the evaluation of all studies involving critically ill patients (MV + nutrition + sepsis). The definitions “MV”, “nutrition” and “sepsis” refer to studies of specific populations. The definition “excluding mortality” refers to the evaluation of other outcomes, in addition to mortality. MV - mechanical ventilation.

For critical illnesses, the outcomes evaluated in RCTs were previously categorized into different domains (Table 1).^(10,15)^ A systematic review by Gaudry et al.^(10)^ evaluated 112 RCTs involving critically ill patients who met the inclusion criteria defined by the authors. The topics most studied were mechanical ventilation (27%), sepsis (19%) and nutrition (17%). The authors identified that patient-centered outcomes were targeted in 65% of the RCTs evaluated. However, when the mortality outcome was excluded from the analysis, only 10% of studies evaluated other patient-centered outcomes, such as quality of life and physical or cognitive performance after discharge from the ICU. Figure 1 shows the prevalence of RCTs whose primary or secondary outcomes were patient-centered, as well as their distribution, based on the focus of the study.

**Table 1:** Categorization of outcomes in intensive care medicine

Domains	Examples
Important outcomes for the patient	Mortality at any time
	Quality of life
	Functional capacity after ICU discharge
	Cognitive capacity after ICU discharge
Clinical outcomes	Organ failure
	Adverse events (e.g., drug-induced skin reaction or hypotension during renal replacement therapy)
	Outcomes associated with medical care (e.g., hospital-acquired pneumonia, catheter-related infections) and delirium
	Clinical events (e.g., venous thromboembolism and myocardial infarction)
	Pain (in the ICU)
	Anxiety (in the ICU)
	Level of consciousness
	Return to spontaneous circulation
	Strength/muscle circumference
	Sleep duration
	NIHSS score for acute phase of CVA
	Clinical response to antibiotics
	Dyspnea (in the ICU)
	Tolerance to noninvasive ventilation
Biological, physiological, or radiological outcomes	BNP
	NGAL
	Total lung capacity
	X-ray severity score
Outcomes related to the decision of the caregiver	Duration of mechanical ventilation
	Length of ICU or hospital stay
	Exposure to antibiotics
	Reintubation
	Exposure to sedation (dose/time)
	Need for renal replacement therapy
	Need for ventilatory support (invasive or noninvasive)
	ICU readmission rate
	Tracheostomy rate
	Need for blood products
	Need for surgical procedure
Outcomes related to care performance	Quality of the care procedure
	Exposure to noise
	Exposure to light
Other outcomes	Family satisfaction
	ICU staff satisfaction
	Costs and charges
	Judgment of the patient on their readiness for discharge
	Team workload
	Compliance with service protocols
	Medico-legal conflict

ICU - intensive care unit; NIHSS - National Institute of Health Stroke Scale; CVA - stroke; BNP - brain-type natriuretic peptide; NGAL - lipocalin associated with neutrophil gelatinase.

More recently, de Grooth et al.^(16)^ demonstrated that intensive medicine journals with a high impact factor have increasingly published studies with primary outcomes focused on disease (e.g., MODS, days without mechanical ventilation, among others). This has occurred since 2016 and in studies with more than 200 - 500 patients. Outcomes focused on diseases are apparently valid but can be interpreted as ambiguous in relation to the real benefit for patients. Therapy can reduce organ failure without improving survival or quality of life, mistakenly suggesting that this new therapy actually benefits the patient.^(16)^ Thus, the choice of disease-focused outcomes, in addition to potentially distant from the wishes of patients, can lead to illusory conclusions regarding the effectiveness of certain treatments. However, notably, when compared to RCTs that target patient-centered outcomes (e.g., mortality), the choice of disease-centered outcomes increase the authors’ chance of finding a positive outcome.^(17,18)^ That is, the sample size calculation is usually smaller because many of the surrogate outcomes are continuous or ordinal variables.

### WHAT OUTCOMES ARE TRULY IMPORTANT FOR PATIENTS?

In clinical research, a relevant outcome for patients has been previously defined as a characteristic or variable that reflects how patients feel, function or survive.^(10,19)^ Clinical studies conducted in diabetic patients have shown that survival, quality of life, and functional, cognitive and neurological performance are the most important outcomes sought by this population.^(20)^ However, when patients are placed in the center of care, the importance of doctors and their opinion decrease in the face of decisions, and they feel capable of making such decisions without the need for further discussion or sharing of expectations. Nevertheless, those most interested in outcomes are those who suffer them: the patients. Physicians and researchers should guide patients on technical issues and on the difficulties in measuring outcomes; however, they must include patients in the discussion and to choose the outcomes to be investigated in RCTs. Only with the participation of more patients in the construction of outcomes would this be possible, placing weight on the “preeminence of the values and preferences of the interested party”.^(21)^ Thus, what outcomes should we measure in critically ill patients?

#### Long-term outcome measures

The study of ICU mortality will always be a marker of care quality.^(15)^ However, the possible adverse consequences of an ICU stay are much better evaluated after discharge from this unit and, especially, after hospital discharge because critical illness is associated with high mortality^(22-24)^ and a high prevalence of long-term adverse effects.^(25-28)^ Approximately 20% of American elderly patients who leave the hospital are readmitted within the first 30 days of discharge.^(29-31)^ Survivors of sepsis have very high mortality in the first months (~40%)^(32)^ and years (~70%)^(23,33)^ after discharge from the ICU. In addition, intensive care survivors experience profound changes in their lives due to the emergence of deficits in one or more domains^(25)^ of physical,^(23,34)^ psychological^(35-37)^ or cognitive functions.^(38)^

Thus, an important decision would be to migrate the evaluation of patients’ outcomes from inside the hospital to outside. This, however, is not yet a reality. Gaudry et al.^(10)^ showed that of the 73 RCTs included in a systematic review evaluating outcomes in critically ill patients, only 17.8% followed the patients for more than 30 days after ICU admission. In addition, the choice of long-term outcomes could hinder the performance and evaluation of clinical studies with critically ill patient populations (Table 2). The higher risk of loss to follow-up may increase selection bias. The high heterogeneity of the instruments used to evaluate patient-centered outcomes in the context of ICU discharge may hinder the adequate summarization and reproducibility of the evidence.^(39)^ For example, in 425 publications examining ICU survivors after hospital discharge, 250 different measurement instruments were identified. Quality of life was the most frequently reported outcome (in 65% of the articles). Physical activity limitations, an outcome that is also highly relevant for patients, appeared in only 6% of the articles. Although this high heterogeneity reflects the growing nature of this research field, it negatively reflects the lack of standardization of measurement instruments,^(8)^ which limits comparisons among studies and hinders the performance of meta-analyses.^(40)^ Finally, many interview instruments have not yet been validated for their application via telephone, a fundamental requirement for the long-term follow-up of patients.

**Table 2:** Challenges for the greater use of long-term and patient-centered outcomes in intensive care

Implementation challenges	Comment
Loss of follow-up	Long follow-up periods may result in large losses to follow-up (due to death or sequela).
Selection bias	More severe patients may become inaccessible over time due to death or severe sequelae, resulting in a population different from that representative of the post-ICU reality.
Memory bias	Long-term follow-up may favor the occurrence of memory bias regarding relevant outcomes, especially if the interval between follow-ups is long.
Confusion bias	Long-term outcomes can be determined by events subsequent to those studied.
Patient-centered outcomes	
The limited inclusion of patients and relatives in the determination of relevant outcomes	There is a gap in the importance of outcomes between patients (and their families) and researchers/health professionals.
Lack of consensus on the health domains evaluated	The lack of consensus among researchers regarding a minimum set of health domains evaluated contributes to the lack of uniformity of publications and a delay in the evolution of knowledge on the subject
High heterogeneity of assessment instruments	The lack of uniformity of measurement instruments can make it difficult to summarize the evidence in meta-analyses, for example

#### Evaluation of mortality associated with quality of life

In the hierarchical distribution of outcomes, mortality always has a prominent role.^(11)^ However, is survival as important for patients as it is for doctors? In studies evaluating the post-ICU life of patients with acute respiratory distress syndrome (ARDS), survival was the outcome best evaluated by researchers and physicians; yet, it was the second least important outcome ranked by patients.^(9)^ This inconsistency could be explained by the study sample, composed only of ICU survivors (obviously not evaluating the deceased); by the propensity of researchers to increase the importance of survival due to their awareness of the importance of accounting for death when evaluating functional outcomes after hospital discharge; and by the common practice of evaluating mortality as the primary outcome in intensive care studies.^(10)^

Unfortunately, surviving critical illness is associated with a wide variety of long-term physical and psychological sequelae that may affect functional status and quality of life.^(25-28)^ Thus, the value of quality of life as a central outcome for ICU survivors is increasing.^(27,41)^ This is an outcome reported by patients themselves, without external interference from researchers or family members. It values the patient’s perspective and allows the evaluation of the real impact of a disease and the consequences of its treatment from a multidimensional aspect (i.e., extrapolating the simple definition of morbidity or mortality). Such multidimensionality makes it possible to evaluate an individual’s perception in relation to different domains of his or her life, such as physical aspects, day-to-day functioning, social performance and emotional aspects.^(27)^ Thus, a good quality of life could increase patient satisfaction more so than determinations of reduced motor capacity or the ability to perform basic or instrumental activities of daily living.

In this view, alone, the survival of a patient who was critically ill does not allow assessing whether he or she recovered his or her happiness, activities, and ability to interact with the environment. Surviving, therefore, does not necessarily mean having quality of life. The authors suggest that both outcomes should always be evaluated together.

#### Combined outcomes of patients, family and/or caregivers

In clinical research environments and long-term follow-up, family members or caregivers are usually informants of the evolution of ICU survivors. However, is patient information reliable? It seems so. In a study conducted with ARDS survivors, researchers and family members were also interviewed.^(9)^ Of the 19 important outcomes selected by the researchers, approximately 80% also showed agreement between patients and their families. The best ranked outcomes were physical function, pulmonary symptoms, cognitive symptoms, mental health symptoms, pain, fatigue, and the ability to return to work or previous activities. Social roles, activities and relationships, survival and sexual symptoms had the lowest levels of agreement. These data highlight that family members serve as substitute informants of patient-centered outcomes (in case of inability to evaluate the patient), aiming to minimize the loss of data related to possible disabilities or the unavailability of patients to answer questionnaires. Notably, family members of critically ill patients usually get sick along with them. Family members experience a high psychological burden in the first year after patient discharge^(42,43)^ as they are suddenly and unpreparedly forced to assume decision-making roles in relation to the conduct and treatment of their loved ones. Thus, considering family members and caregivers as a population that should have their outcomes studied seems logical, in addition to the fact that keeping them in clinical follow-up could bring them benefits.^(44)^

### BARRIERS TO IMPLEMENTING CHANGE

Change is difficult to accept in any field of science, be it exact or social.^(45)^ The trade of a “paternalistic model” of medical decision-making to a “model of sharing” decisions with patients (in which the “patient is at the center” of medical decision-making) has been changing the scale of importance of the outcomes studied. It seems that now there is less interest in what is a “clinically relevant” effect and more emphasis on what is “important for the patient”.^(16,46,47)^

Some difficulties are expected as this slow change occurs in scientific studies of critically ill patients. First, to date, there is no taxonomy of the outcomes studied in critically ill patients or a defined grouping of the set of outcomes.^(10,40,48,49)^ However, some experiments are already being performed in some medical specialties, such as rheumatology^(50,51)^ and endocrinology,^(20)^ and in patient education studies.^(19)^ Additionally, in the area of intensive care, initiatives in the areas of ventilatory support^(52)^ and acute respiratory failure^(53-55)^ have emerged.

Second, the discovery of how different the expectations of patients and physicians are regarding the outcomes proposed in studies is relatively recent,^(9,12,13,56,57)^ that is, still too early to be common knowledge among the entire medical community.

Third, methods to involve patients in determining patient-centered outcomes are still under development and include conducting qualitative research.^(58)^ as well as the need for patient participation in health-related meetings or conferences.^(8,40,57)^ To date, few medical conferences allow active participation of patients in discussion sessions.

Fourth, opting for primary outcomes focused on disease (e.g., oxygenation index, organ failure score, shock reversal time, and ventilation-free days, among others) usually requires a smaller sample size and may be a more sensitive indicator of the effects of a given treatment, when compared to truly important outcomes (such as survival or quality of life).^(16)^

Finally, the choice of a composite outcome (a disease-centered outcome associated with a patient-centered outcome) to facilitate the execution of a study could be difficult to interpret because the treatment offered often has different effects on each individual components of the outcome.^(16)^ This fact could greatly complicate the interpretation of this “new” composite outcome proposed for studies.

## FINAL CONSIDERATIONS

Disease-centered primary outcomes have become more prevalent in intensive care studies. The choice of patient-centered outcomes would correct the wrong course of current medical research. However, numerous financial, organizational, and individual barriers prevent this “correct” transition to occur as quickly as it should. The choice of an outcome in a scientific study should be built in collaboration, in which the patient, family, researcher and clinician perspectives are evaluated, discussed, and synthesized to obtain a cohesive and representative understanding of the results that would be important for the patients, in addition of being easy to perform and interpret by physicians, relatives and researchers.
